# Aircraft Engine Prognostics Based on Informative Sensor Selection and Adaptive Degradation Modeling with Functional Principal Component Analysis †

**DOI:** 10.3390/s20030920

**Published:** 2020-02-09

**Authors:** Bin Zhang, Kai Zheng, Qingqing Huang, Song Feng, Shangqi Zhou, Yi Zhang

**Affiliations:** 1 School of Advanced Manufacturing Engineering, Chongqing University of Posts and Telecommunications, Chongqing 400065, China; zhangbin@cqupt.edu.cn (B.Z.); zhengkai@cqupt.edu.cn (K.Z.); fengsong@cqupt.edu.cn (S.F.); zsq11915@163.com (S.Z.); zhangyi@cqupt.edu.cn (Y.Z.); 2 The State Key Laboratory of Mechanical Transmissions, Chongqing University, Chongqing 400044, China; 3 School of Automation, Chongqing University of Posts and Telecommunications, Chongqing 400065, China

**Keywords:** prognostics, aircraft engine, sensor selection, degradation modeling, functional principal component analysis, Bayesian inference

## Abstract

Engine prognostics are critical to improve safety, reliability, and operational efficiency of an aircraft. With the development in sensor technology, multiple sensors are embedded or deployed to monitor the health condition of the aircraft engine. Thus, the challenge of engine prognostics lies in how to model and predict future health by appropriate utilization of these sensor information. In this paper, a prognostic approach is developed based on informative sensor selection and adaptive degradation modeling with functional data analysis. The presented approach selects sensors based on metrics and constructs health index to characterize engine degradation by fusing the selected informative sensors. Next, the engine degradation is adaptively modeled with the functional principal component analysis (FPCA) method and future health is prognosticated using the Bayesian inference. The prognostic approach is applied to run-to-failure data sets of C-MAPSS test-bed developed by NASA. Results show that the proposed method can effectively select the informative sensors and accurately predict the complex degradation of the aircraft engine.

## 1. Introduction

As the heart of an aircraft, the engine consists of several subsystems with millions of parts, and its health condition directly impacts operation and safety of the whole aircraft system. While the reliability of aero-engines has been improved over the years, factors such as fatigue and wear will inevitably cause the health condition of an engine to degrade with usage, reducing the overall performance of the powered aircraft [1]. Therefore, a number of sensors of varying types have been widely used to monitor the health degradation of aircraft engines, which creates a multi-sensor environment for operational health analysis and maintenance decision-making. Over the past decades, prognostics that utilizes monitoring sensors information to predict the future health and estimates the remaining useful life (RUL) before failure/end-of-life has attracted increasing attention from academic researchers and industrial operators [2]. For its potential in enhancing reliability and maintenance efficiency while reducing unnecessary maintenance and minimizing operational costs, prognostics have been an active research field for aircraft engine applications [3].

Generally, the existing methods pertaining to aircraft engine prognostics can be classified into two categories: model-based and data-driven methods. For model-based methods, a mathematical model that can describe the engine health degradation process that is normally required to be constructed according to physical failure characteristics before prediction. Some typical model-based methods have been developed for engine health estimation and RUL prediction, such as the Markov model-based [4,5] and particle filtering-based method [6,7]. While model-based methods enable improved accuracy of engine prognostics, certain assumptions and simplifications of the adopted models may pose limitations on their practical deployment. With the rapid development of data mining techniques and the growing availability of health monitoring data, data-driven methods attract increasing attention. Data-driven methods utilize the information extracted or learned from observed data to identify the degradation behavior and predict the future health condition without using any particular physical model [8,9]. In this view, data-driven methods may be the more applicable prognostic solution for complicated systems, as aero-engines that have limited knowledge of physics-of-failure but have massive multi-sensor monitoring data. Among the data-driven prognostic methods, machine learning and statistical approaches are two popular branches. The common machine learning techniques used for prognostics include support vector regression [10], artificial neural network [11,12], fuzzy logic [13], etc. Machine learning methods are capable of dealing with prognostic issues of complex systems, but the predicted results are hard to be explained because of the lack of transparency. Besides, it is difficult for machine learning methods to handle the uncertainty in health degradation and long-term prognostics. In contrast, statistical approaches fit probabilistic models to the health monitoring data and are effective in managing the uncertainty of the degradation process as well as its influence on future predictions. Thus, statistical prognostics are recently becoming dominant [2]. 

In statistical data driven prognostics, the available health monitoring data that contains useful health degradation information is modeled by statistical models to perform prognostics [14]. Initially, regression-based statistical models are applied to model the available data and predict future health. In [15], a hybrid multi-variate adaptive regression splines method was proposed for the aircraft engine prognosis. To account unit-to-unit variability, Zaidan et al. [16] formulated a Bayesian hierarchical model with linear random coefficient model for engine prognostics. A parametric *p*th order polynomial model was proposed by Liu et al. [17,18] and was validated on a degradation dataset of an aircraft gas turbine engine. Another group of frequently used statistical models are stochastic process models that can well capture the degradation dynamics of a system. For its nice mathematical properties, the Wiener process has been intensively studied in degradation modeling and prognostics for engines and other engineered systems [19]. Considering monotone non-decreasing degradation in practice, Le Son et al. [20] modeled deterioration of gas turbine engines as a non-homogeneous Gamma process. Peng et al. [21] further researched modeling of time-varying degradation rates with an inverse Gaussian process. In practice, statistical approaches are mainly concentrated on univariate degradation modeling and prognostics, so a composite health index (HI) that captures health degradation of an engine is normally needed to be constructed by fusion of the multi-sensor monitoring data. Wang et al. [22] proposed a HI construction method by a linear data transformation method of multi-sensor data. In [23], the failure time and the multi-sensor data are combined via a novel latent linear model to construct a generic HI. Theoretically, integration of more sensors may be beneficial for prognostics. However, different sensors usually have different levels of relevance to the degradation process. Also, some sensors may be even unrelated to the underlying degradation process due to high noise, low sensitivity to the health degradation, inconsistency among the population, etc. In addition, fusion of too many sensors may incur a heavy data processing burden and unnecessary costs. As a result, it is better to select the degradation-relevant sensors for HI construction, and hereafter, this kind of sensors are denoted as informative sensors in this paper. Coble et al. [24] proposed three metrics to characterize the suitability of sensors for prognostics. Yu [6] proposed an informative sensor selection method based on logistic regression with penalization regularization for aircraft engine health prognostics. In [25], Liu et al. proposed an improved permutation entropy method for selection of informative sensors. To identify multiple stages before failure, Chehade et al. studied informative sensor selection and fusion using statistical hypothesis testing [26]. Besides, the task of informative sensor selection was integrated with the statistical modeling in [23] and [27] for engine prognostics. 

Through fusion of the selected informative sensors into a composite HI, many statistical methods have been proposed for prognostics of complex systems, including aircraft engines. However, most of the statistical approaches are based on parametric models, such as a linear model [16,23,28], exponential model [17,27], quadratic model [24], etc. These methods are effective when the engine degradation pattern can be described with a specific model, but there are numerous cases where the degradation trajectory may not be appropriately fitted by a parametric model [1,3,16]. Therefore, a more flexible degradation modeling and prognosis technique is needed for practical applications. Furthermore, the existing literature relevant to the selection of informative sensors is basically focused on the relevance of a sensor to the degradation process. While metrics of monotonicity [10,25], correlation and robustness [2,29] have been commonly utilized to evaluate the relevance of sensors for prognostics. There is a lack of efforts to consider the degree to which the sensors of a population of systems have the same underlying shape [24]. Specifically, for a fleet of engines operating under one operational condition and with one failure mode, it is highly desired that the sensor demonstrating a consistently increasing or decreasing trend for all the engines to be selected as the informative sensor. To address these limitations, a statistical method with informative sensor selection is proposed for prognostics of the aircraft engine in this paper. In the presented method, a new metric that evaluates the degree of consistent trend of a sensor for a population of systems is proposed and used to select informative sensors to construct the engine HI. Then, the functional principal component analysis (FPCA) method is applied to adaptively model the engine HI and combined with Bayesian inference for future prognostics.

The remainder of this paper is organized as follows. Section 2 briefly gives the related theories. Section 3 presents a detailed description of the proposed prognostic approach. Section 4 demonstrates the developed method with a case study on the degradation of a turbofan engine. Section 5 discusses some key issues of the proposed approach. Finally, Section 6 summaries the research and future work.

## 2. Related Theories

In this section, the theories utilized in this study are briefly introduced.

### 2.1. Basics of FPCA

Functional data analysis concerns the analysis of data that are in the form of a function with statistical methods. Functional data are of intrinsically infinite dimension, and thus, dimension reduction based on some kind of basis function is crucial for analysis of such data. Unlike the parametric basis function such as B-spline, Fourier, and wavelet functions, FPCA directly derives the basis function from the data and has been a useful tool for functional data analysis [30,31,32].

In functional data analysis, the given data *Y_i_*(*t*) (*i* = 1, 2, …, *n*) is assumed as an independent and identically distributed (i.i.d) realization of a stochastic process Y(*t*) that is in *L*^2^ and defined on the interval [0, *Γ*]. The expectation and covariance function of Y(*t*) are respectively *μ*(*t*) = *E*(Y(*t*)) and *G*(*s*, *t*) = *E*((Y(*s*) − *μ*(*s*))(Y(*t*) − *μ*(*t*))), where *E*(·) is the expectation operator. Under mild assumptions, Mercer’s theorem implies that the spectral decomposition of *G*(*s*, *t*) leads to:

(1) $$G\left(s,t\right)=\sum_{k=1}^{\infty }\lambda_{k}\phi_{k}\left(s\right)\phi_{k}\left(t\right),\;t\in \left[0,\Gamma \right]$$

where *λ*_1_ ≥ *λ*_2_ ≥ ... are the ordered eigenvalues and *ϕ_k_*(*t*) are the corresponding eigenfunctions or functional principal components (FPCs). These terms are obtained by solving the following eigen-decomposition problem:

(2) $$\begin{array}{c} \int_{\Gamma }G\left(s,t\right)\phi_{k}\left(t\right)ds=\lambda_{k}\phi_{k}\left(t\right),\;t\in \left[0,\Gamma \right]\\ s.t.\left\{ \begin{array}{l} \int_{\Gamma }\phi_{k}{}^{2}\left(t\right)dt=1\\ \int_{\Gamma }\phi_{l}\left(t\right)\phi_{k}\left(t\right)dt=0,l\neq k \end{array}\right. \end{array}$$

Then the Karhunen–Loève theorem states that the functional data *Y_i_*(*t*) (*i* = 1, 2, …, *n*) are represented by FPCs of the underlying stochastic process covariance function as Equation (3). In the statistical literature, this method has been coined FPCA:

(3) $$Y_{i}\left(t\right)=\mu \left(t\right)+\sum_{k=1}^{\infty }\varepsilon_{ik}\phi_{k}\left(t\right),\;t\in \left[0,\Gamma \right]$$

where *ε_ik_* are the FPC scores calculated as Equation (4), and *ε_ik_* are random variables that are independent across *i* and uncorrelated across *k* with *E*(*ε_ik_*) = 0 and *E*(*ε_ik_*^2^) = *λ_k_*:

(4) $$\varepsilon_{ik}=\int_{\Gamma }\left(Y_{i}\left(t\right)-\mu \left(t\right)\right)\phi_{k}\left(t\right)dt,\;t\in \left[0,\Gamma \right]$$

Based on the above analysis, the functional data is expressed as the sum of infinite terms of FPCs by FPCA. Each FPC independently depicts one mode of variations in the functional data, thus the variance (*Var*) of the data *Y_i_*(*t*) (*i* = 1, 2, …, *n*) is the sum of all the eigenvalues of the covariance function as follows:

(5) $$Var\left(Y_{i}\left(t\right)\right)=Var\left(Y_{i}\left(t\right)-\mu \left(t\right)\right)=\sum_{k=1}^{\infty }Var\left(\varepsilon_{ik}\right)=\sum_{k=1}^{\infty }\lambda_{ik}$$

However, only a small number of eigenvalues are commonly significantly nonzero in practice. For eigenvalues which are approximately zero, the corresponding FPC scores will also be approximately zero. Therefore, *Y_i_*(*t*) (*i* = 1, 2, …, *n*) can be approximated by the first *K*-truncated terms as:

(6) $$Y_{i}\left(t\right)=\mu \left(t\right)+\sum_{k=1}^{K}\varepsilon_{ik}\phi_{k}\left(t\right),\;t\in \left[0,\Gamma \right]$$

To ensure that sufficient variance of the functional data is explained by the truncated expansion, *K* is usually chosen using the fraction of variance explained (FVE) method as Equation (7) and the threshold *η* is often a value higher than 85% [30]:

(7) $$K=\underset{k}{\min}\left(\frac{\sum_{i=1}^{k}\lambda_{i}}{\sum_{i=1}^{\infty }\lambda_{i}}\geq \eta \right)$$

### 2.2. Bayesian Inference

Bayesian inference is based on the Bayes’ theorem, which allows one to formally incorporate prior knowledge or experience into computing statistical probabilities. In this way, the inference takes the accumulated information as a priori, and then uses the observed data to update the priori to a posterior. Currently, Bayesian inference has been widely applied in reliability engineering [8,16,21]. 

For a continuous random variable *y* and related random parameters **θ**, Bayes’ theorem states that their joint probability can be written in two ways as [33]:

(8) p(θ,y)=p(y|θ)p(θ)=p(θ|y)p(y)

Eliminating the joint probability *p*(*y*, **θ**) and rearranging a bit, we obtain Bayesian inference for the parameters **θ**:

(9) $$p\left(\theta |y\right)=\frac{p\left(y|\theta \right)p\left(\theta \right)}{p\left(y\right)}\propto p\left(y|\theta \right)p\left(\theta \right)$$

In the literature, *p*(**θ**|*y*) and *p*(**θ**) are respectively defined as the posterior and prior distribution of the random parameters **θ**, and *p*(*y*|**θ**) is denoted as the likelihood function. The *p*(*y*|**θ**) can be understood as the probability of observing samples of the random variable *y* conditioned on the parameters **θ**. Based on Equation (9), Bayesian inference updates the parameters distribution from a priori to a posterior with the likelihood function of the observed data. Thus, the uncertainty in the parameters can be reduced and accuracy of further inference using the parameters can be improved.

## 3. The Proposed Method

**Figure 1:**
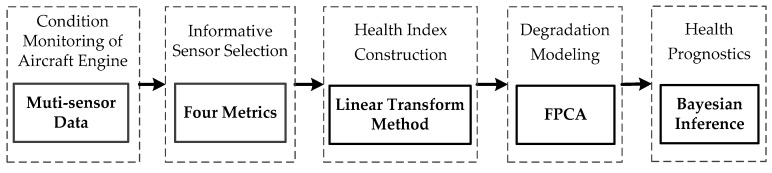
Flowchart of the proposed method. Abbreviations: FPCA, functional principal component.

This section is devoted to introducing the proposed method. The prognostic approach consists of informative sensor selection, HI construction, degradation modeling, and health prognostics. Flowchart of the method is shown in Figure 1.

### 3.1. Informative Sensor Selection

In this subsection, four metrics are presented to select informative sensors for complex systems of aircraft engines with a number of monitoring sensors.

In practice, system health deterioration is inherently a stochastic process. Metrics for informative sensor selection should be defined upon separating a sensor measurement into its trend and the residual. This decomposition can be carried out locally with smoothing methods or globally with parametric or nonparametric methods. To make full use of the sensor measurements for the whole degrading process modeling and to preserve local characteristics of the degradation, local smoothing methods may be a better option. In this paper, the trend of a sensor measurement is obtained using the moving average method. With the sensor trend and residual, three metrics for degradation feature selection were defined in [29] to improve prognostics effectiveness and efficiency. For the sake of completeness and clarity, the three metrics are reformulated for sensor selection as:

(10) $$\begin{array}{l} s\left(t_{j}\right)=s_{T}\left(t_{j}\right)+s_{R}\left(t_{j}\right),Corr\left(S\right)=\frac{\left|N\sum_{j}s_{T}\left(t_{j}\right)t_{j}-\sum_{j}s_{T}\left(t_{j}\right)\sum_{j}t_{j}\right|}{\sqrt{\left[N{\sum_{j}s_{T}\left(t_{j}\right)}^{2}-{\left(\sum_{j}s_{T}\left(t_{j}\right)\right)}^{2}\right]\left[N{\sum_{j}t_{j}}^{2}-{\left(\sum_{j}t_{j}\right)}^{2}\right]}}\\ Mon\left(S\right)=\frac{\left|\sum_{j}\delta \left(s_{T}\left(t_{j}\right)-s_{T}\left(t_{j-1}\right)\right)-\sum_{j}\delta \left(s_{T}\left(t_{j-1}\right)-s_{T}\left(t_{j}\right)\right)\right|}{N-1},Rob\left(S\right)=\frac{1}{N}\sum_{j}\exp\left(-\left|\frac{s_{R}\left(t_{j}\right)}{s_{T}\left(t_{j}\right)}\right|\right) \end{array}$$

where *s*(*t_j_*) is the measurement of the sensor *S* at the time *t_j_* (*j* = 1, 2, …, *N_j_*) with the trend of *s_T_*(*t_j_*) and residual *s_R_*(*t_j_*), *δ*(·) is the simple unit step function.

Among the three metrics, correlation (Corr) measures the linearity between the interested sensor and the usage time, monotonicity (Mon) assesses consistently increasing or decreasing trend of the sensor, and robustness (Rob) reflects the tolerance of the sensor to noises and outliers. The sensor with higher scores should be selected as the informative sensor, for the three metrics are all positively correlated with the relevance of a sensor to the system degradation. 

From Equation (10), it can be concluded that these metrics are defined on the sensor from one individual system and the same sensor from other systems of the population is not accounted. For a population of systems (such as a group of aircraft engines) that operate under the same condition and with one failure mode, the informative sensor is highly desired to have a consistent increasing or decreasing trend for all the systems. Additionally, the sensor with a small variation of measurements upon system failures and a large range till failure is also desired for prognostics. Thus, a new metric of predictability (Pre) is formulated for sensor selection as:

(11) $$Pre\left(S\right)=Exp\left(-\frac{std\left(s_{f}\right)}{\left|mean\left(s_{f}\right)-mean\left(s_{s}\right)\right|}\right)$$

where *s_f_* and *s_s_* are respectively the measurements at the failure and start instant of the sensor *S* for a population of systems.

The new metric Pre takes the failure and start values of a sensor from the entire population into account, and thus the trend consistency of a sensor among a population is given consideration. Also, Pre is positively related to the performance of the sensor with the range of [0,1], and favorites the sensor with well-clustered failure values and large range. That is the sensor with a high score of Pre should be selected as the informative sensor. 

For prognostic parameter selection, Coble et al. presented three metrics in [24] and the prognosability (Pro) metric is quite similar to the proposed Pre. However, the two metrics are different in measuring the range of one sensor. In the metric Pro, the range is the mean range from start to failure for a population of systems. Sensors with well-clustered failure values and large range are encouraged by this measure. While in the Pre, as in Equation (11), the sensor measurements at the start and failure instant of a population are respectively averaged to the given range of the sensor. The metric Pre selects sensors with both well-clustered failure and start values as well as large range.

To select the informative sensor for aircraft engines, two steps are included using the presented goodness metrics. In the first step, binary-value and constant-value sensors are eliminated by simple visual inspection, because these sensors do not make sense for prognosis. As can be seen from these definitions, one goodness metric only partially measures suitability of a sensor for prediction and sensor selection based on only one metric will be biased. In the second step, sensors are evaluated by four metrics simultaneously, and then the informative sensors selected with drop out strategy are fused to construct a HI for health prognosis of aircraft engines.

### 3.2. Health Index Construction

For a data-driven prognostics, extraction of the health signatures and background knowledge from massive training/testing sensors is required. Based on the selected informative sensors, the HI construction is considered in this subsection. 

The transformation of the selected informative sensors into one-dimensional HI is a process of information fusion, which enables a general measure to characterize the health condition of a system and also the degradation of different systems from the same population to be similar. The HI can be constructed using many information fusion techniques, such as linear data transformation method [13,17,22], PCA and Euclidean distance measure [20], and logistic regression [6,11]. For its effectiveness and ease of use, the linear data transformation method is also employed to construct the HI for aero-engines in this paper.

Suppose the selected informative sensors are *d*-dimensional and the two sensor data sets that represent the aircraft engine failed, and healthy states are **Q**_0_ of *M*_0_ × *d* and **Q**_1_ of *M*_1_ × *d* matrix, where *M*_0_ and *M*_1_ are, respectively, the data sizes for engine failed and healthy states. Generally, data sets for healthy and failed states can be collected from the training data sets at the beginning and at the end of the run-to-failure tests. With these two data sets, a transformation matrix **T** of *d* × 1 is obtained and then the one-dimensional HI **y** for any *d*-dimensional matrix **Q** is:

(12) $$\begin{array}{l} y=QT\\ T={\left(Q_{off}{}^{T}Q_{off}\right)}^{-1}Q_{off}{}^{T}S_{off} \end{array}$$

where **Q_*off*_** = [**Q_0_**; **Q_1_**]^T^, **S_*off*_** = [**S_0_**, **S_1_**]^T^, **S_0_** is a 1 × *M*_0_ zero vector and **S_1_** is a 1 × *M*_1_ unity vector.

As can be easily derived, the HI obtained by the linear transformation method as Equation (12) is varying approximately between 1 and 0. With the usage of the aircraft engine, the HI has a decreasing trend. This HI contains health condition information extracted from multi-dimensional informative sensors of the monitored aircraft engine. It can be used to construct background health knowledge in the offline process and to further conduct the online prediction process. Although the linear transformation is discussed here and will be employed for the case studies, other information fusion methods can also be used to construct the HI with the selected informative sensors.

### 3.3. Degradation Modeling by FPCA

In view that the health degradation is a stochastic process with uncertainties from physical degradation dynamics, usage variations and other effects, the degradation pattern of an aircraft engine is complex and unknown. To adaptively study the aircraft engine degradation, the HI that describes the health deterioration of an engine is assumed as discrete sampling values of the functional data and is modeled with the FPCA method in this subsection.

Suppose there is a population of aircraft engines, then the degradation pattern of one aircraft engine is an independent realization of the degradation process of the whole population. Based on the FPCA analysis method briefly introduced in Section 2, the degradation of one engine can be represented by the mean function and FPCs derived adaptively from the population stochastic degradation process X(*t*) (*t*∈*I*), where *I* is the service time interval of the population of engines (the observation interval for the engine/system with the longest possible lifetime). Further, considering the fact that the HI indirectly demonstrates the health degradation of the engine, so the measurement error is one important aspect that should be accounted for. To be specific, for a population of *n* aircraft engines with *N_i_* measurements for the *i*-th engine, the degradation of the *i*-th engine is modeled with its HI *y_i_*(*t_ij_*) (*i* = 1, 2, …, *n*; *j* = 1, 2, …, *N_i_*) by FPCA as:

(13) $$\begin{array}{l} y_{i}\left(t_{ij}\right)=x_{i}\left(t_{ij}\right)+e_{ij}\\ x_{i}\left(t\right)=\mu \left(t\right)+\sum_{k=1}^{K}\varepsilon_{ik}\phi_{k}\left(t\right) \end{array}$$

where *x_i_*(*t_ij_*) and *e_ij_* are the unobservable degradation and measurement error of the *i*-th engine at time *t_ij_*, *x_i_*(*t*) is the underlying functional data with the mean function *μ*(*t*) and the FPCs *ϕ_k_*(*t*)s for the *i*-th engine, and *ε_ik_* are the related FPC scores.

With the FPCA-based degradation modeling as Equation (13), the degradation characteristics of the whole engine population is represented by the mean function and the first *K* FPCs, while the degradation peculiarity of an engine is captured by its specific FPC scores. The mean function *μ*(*t*) describes the common degradating trend of all the engines from an engine population, and the first few FPCs reflect the main varying modes of the population degradation process. As in [27,34], the measurement errors *e_ij_* are practically assumed to be i.i.d with the normal distribution *N*(0, *σ*^2^) and are also independent of FPC scores in this study. Also, note that no pre-specified parametric form is needed to be assumed for *μ*(*t*) and the *ϕ_k_*(*t*)s, but they are adaptively derived from the engines HI in the following.

For the mean function *μ*(*t*) = *E*(X(*t*)), it is estimated by local linear smoothers using degradation observations *y_i_*(*t_ij_*) (*i* = 1, 2, …, *n*; *j* = 1, 2, …, *N_i_*) from all *n* engines as:

(14) $$\begin{array}{l} \hat{\mu }\left(t\right)=\alpha_{0}\left(t\right)\\ \underset{\alpha_{0},\alpha_{1}}{\min}\sum_{i}\sum_{j}\kappa_{1}\left(\frac{t_{ij}-t}{h_{\mu }}\right){\left(y_{i}\left(t_{ij}\right)-\alpha_{0}-\alpha_{1}\left(t-t_{ij}\right)\right)}^{2} \end{array}$$

where κ_1_(·) is a univariate kernel function and *h_μ_* is the smoothing bandwidth.

For estimations of the *ϕ_k_*(*t*)s, the covariance function *G*(*s*, *t*) = *E*((X(*s*) − *μ*(*s*))(X(*t*) − *μ*(*t*))) of the engine degradation process X(*t*) should be firstly estimated. For the *n* engines, the following holds:

(15) $$\begin{array}{l} V_{i}\left(t_{ij},t_{il}\right)=\left(y_{i}\left(t_{ij}\right)-\hat{\mu }\left(t_{ij}\right)\right)\left(y_{i}\left(t_{il}\right)-\hat{\mu }\left(t_{il}\right)\right)\\ E\left(V_{i}\left(t_{ij},t_{il}\right)\right)=V\left(t_{ij},t_{il}\right)=G\left(t_{ij},t_{il}\right)+\sigma^{2}\delta_{jl} \end{array}$$

where *δ_ij_* is the Kronecker delta function.

It is easy to see that the diagonal (i.e., *j* = *l*) of the raw covariance function *V*(*t_ij_*, *t_il_*) calculated with the degradation observations are contaminated with measurement errors. Estimation of the covariance function *G*(*s*, *t*) using the local linear surface smoothing should be without the diagonal as:

(16) $$\begin{array}{l} \hat{G}\left(s,t\right)=\beta_{0}\left(s,t\right)\\ \underset{\beta_{0},\beta_{11},\beta_{12}}{\min}\sum_{i}\sum_{1\leq j\neq l\leq N_{i}}\kappa_{2}\left(\frac{t_{ij}-s}{h_{G}},\frac{t_{il}-t}{h_{G}}\right){\left(V_{i}\left(t_{ij},t_{il}\right)-\beta_{0}-\beta_{11}\left(s-t_{ij}\right)-\beta_{12}\left(t-t_{il}\right)\right)}^{2} \end{array}$$

where κ_2_(·) is a bivariate kernel function and *h_G_* is the smoothing bandwidth.

Then eigen-decomposition following Equation (2) by discretizing the $\hat{G}\left(s,t\right)$ is performed to obtain the eigenvalues ${\hat{\lambda }}_{k}$ (*k* = 1, 2, …) and the corresponding FPCs. Spline interpolation is further utilized to obtain the continuous ${\hat{\phi }}_{k}(t)$s.

For the variance *σ*^2^ of the measurement errors, it is related to the diagonal values of *V*(*t_ij_*, *t_il_*) and *G*(*s*, *t*). To mitigate boundary effects, its estimation is:

(17) $${\hat{\sigma }}^{2}=\max\left(\frac{2}{ℐ}\int_{{ℐ}_{1}}\left(\hat{V}\left(t\right)-\tilde{G}\left(t\right)\right)dt,0\right)$$

where $\hat{V}\left(t\right)$ is the local linear smoother of diagonal values of *V*(*t_ij_*, *t_il_*), $\tilde{G}\left(t\right)$ is the diagonal values of $\hat{G}\left(s,t\right)$, and the interval *I*_1_ = [*I*/4, 3*I*/4]. 

Considering the measurement errors in the degradation model, to estimate FPC scores with Equation (4) will lead to bias. To remedy that, the conditional expectation method proposed by Yao et al. [35] is utilized here to estimate the *ε_ik_*:

(18) $${\hat{\varepsilon }}_{ik}={\hat{\lambda }}_{k}{\hat{\phi }}_{ik}^{T}{\hat{\Sigma }}_{i}^{-1}\left(y_{i}-{\hat{\mu }}_{i}\right)$$

where **y***_i_* = (*y_i_*_1_, *y_i_*_2_, ..., *y_iNi_*)*^T^* is the engine HI observing vector, ${\hat{\mu }}_{i}={\left({\hat{\mu }}_{i1},{\hat{\mu }}_{i2},\ldots ,{\hat{\mu }}_{iNi}\right)}^{T}$ and ${\hat{\phi }}_{i\kappa }={\left({\hat{\phi }}_{i1},{\hat{\phi }}_{i2},\ldots ,{\hat{\phi }}_{iNi}\right)}^{T}$ are the mean and FPC vectors interpolated from the mean function and FPCs, and ${\hat{\Sigma }}_{i}$ is with the entry (*j*, *l*) as ${\hat{\Sigma }}_{i}\left(j,l\right)=\hat{G}\left(t_{ij},t_{il}\right)+{\hat{\sigma }}^{2}\delta_{jl}$.

With the estimated parameters, the uncontaminated degradation of the *i*-th engine is modeled by FPCA as:

(19) $${\hat{x}}_{i}\left(t_{ij}\right)=\hat{\mu }\left(t_{ij}\right)+\sum_{k=1}^{K}{\hat{\varepsilon }}_{ik}{\hat{\phi }}_{k}\left(t_{ij}\right)$$

Based on the FPCA modeling analysis, degradation of an engine is statistically represented with the common trend and a few varying terms that reflect the main dynamics of the degradation process. For estimation of the model parameters, all degradation observations of the engine population are fused and local linear smoothing is needed. In this study, the Gaussian kernel function is applied for both curve and surface smoother, and the related smoothing bandwidth is determined by the one-leave-out cross-validation strategy. 

### 3.4. Prognostics with Bayesian Inference

For one aero-engine that is still operating, its future health is of significance for condition-based maintenance and health management. Therefore, Bayesian inference is combined with the degradation model for engine prognostics in this Subsection.

With the degradation modeling by FPCA as detailed in the previous Subsection, the degradation trajectory of one in-service engine can be described as:

(20) $$x\left(t\right)=\mu \left(t\right)+\sum_{k=1}^{K}\varepsilon_{k}\phi_{k}\left(t\right)$$

Assume that we have monitored the in-service engine at a vector of time **t** = (*t*_1_, *t*_2_, ..., *t_H_*) with the observed HI **y**_1:*H*_ = (*y*_1_, *y*_2_, ..., *y_H_*)*^T^*, where *t_H_* denotes the latest monitoring time. Once the mean function *μ*(*t*) and FPCs *ϕ_k_*(*t*)s are estimated with HI of historical run-to-failure engines of the population as elaborated in Section 3.3, the trend and the main variations of the in-service engine degradation can be obtained by interpolations with its monitoring times. However, the FPC scores **ε** = (*ε*_1_, *ε*_2_, ..., *ε_K_*)*^T^* are to be inferenced.

In practice, system health deterioration is usually a gradual process under the effects of various internal and external environmental factors, and the degradation process can be assumed as a Gaussian process. Then the uncorrelated FPC scores *ε_k_* (*k* = 1, 2, ..., *K*) will respectively follow the normal distributions *N*(0, *λ_k_*) with *λ_k_* being the ordered eigenvalues of the covariance function of the degradation process. Taking FPC scores estimated from the historical run-to-failure engines as the prior distribution *p*_0_(**ε**) and considering the likelihood function *p*(**y**_1:*H*_|**ε**) of observing **y**_1:*H*_ [36], we have:

(21) $$\begin{array}{l} p_{0}\left(\varepsilon \right)∼N\left(0,\Lambda \right),\Lambda =diag\left({\hat{\lambda }}_{1},{\hat{\lambda }}_{2},\ldots ,{\hat{\lambda }}_{K}\right)\\ p\left(y_{1:H}|\varepsilon \right)=\frac{1}{\sqrt{2\pi {\hat{\sigma }}^{2}}}\exp\left(-\frac{e^{T}e}{2{\hat{\sigma }}^{2}}\right),e=y_{1:H}-\mu_{1:H}-\Phi \varepsilon \end{array}$$

where **μ**_1:*H*_ and **Φ** with entry (*h*, *k*) as ${\hat{\phi }}_{k}(t_{h})$ (*h* = 1, 2, …, *H*; *k* = 1, 2, …, *K*) are respectively interpolated with the monitoring time vector **t** = (*t*_1_, *t*_2_, ..., *t_H_*) from the $\hat{\mu }\left(t\right)$ and ${\hat{\phi }}_{k}(t)$s estimated from the run-to-failure engines, and ${\hat{\sigma }}^{2}$ is the estimated measurement error.

Based on Bayesian inference outlined in Section 2.2, the posterior distribution *p*(**ε**|**y**_1:*H*_) of the FPC scores **ε** of the in-service engine can be updated as:

(22) $$p\left(\varepsilon |y_{1:H}\right)\propto p_{0}\left(\varepsilon \right)p\left(y_{1:H}|\varepsilon \right)$$

From Equation (21), the *p*_0_(**ε**) and *p*(**y**_1:*H*_|**ε**) are conjugate multivariate normal distributions. The *p*(**ε**|**y**_1:*H*_) can be analytically derived to also follow multivariate normal distribution as follows:

(23) $$\begin{array}{l} p\left(\varepsilon |y_{1:H}\right)∼N\left(\mu_{H}^{\varepsilon },\Sigma_{H}^{\varepsilon }\right)\\ \Sigma_{H}^{\varepsilon }={\left(\frac{\Phi^{T}\Phi }{{\hat{\sigma }}^{2}}+\Lambda^{-1}\right)}^{-1},\mu_{H}^{\varepsilon }=\Sigma_{H}^{\varepsilon }\frac{\Phi^{T}}{{\hat{\sigma }}^{2}}\left(y_{1:H}-\mu_{1:H}\right) \end{array}$$

Further inserting the posteriori distribution of the FPC scores **ε** into Equation (20), a real-time prognosis for the in-service engine based on the latest observations **y**_1: *H*_ is:

(24) $$\begin{array}{l} x\left(t\right)∼N\left(\mu_{H}^{x}\left(t\right),\sigma_{H}^{2x}\left(t\right)\right)\\ \mu_{H}^{x}\left(t\right)=\mu \left(t\right)+\phi^{Τ}\left(t\right)\mu_{H}^{\varepsilon },\sigma_{H}^{2x}\left(t\right)=\phi^{Τ}\left(t\right)\Sigma_{H}^{\varepsilon }\phi \left(t\right) \end{array}$$

where $\mu \left(t\right)=\hat{\mu }\left(t\right)$ and $\phi \left(t\right)={\left({\hat{\phi }}_{1}\left(t\right),{\hat{\phi }}_{2}\left(t\right),\ldots ,{\hat{\phi }}_{K}\left(t\right)\right)}^{T}$ are respectively the mean value and FPC vector interpolated from the mean function and *K* FPCs at a future time *t*.

## 4. Case Studies and Results Analysis

The main purpose of this section is to demonstrate the validity and performance of the proposed prognostics approach with the case study on an aircraft gas turbine engine.

### 4.1. Sensor Data of Aircraft Engine

The sensor data of the aircraft gas turbine engine degradation is generated from the commercial modular aero-propulsion system simulation (C-MAPSS) developed at NASA [37] and published online for research investigations. Each time series signal represents a different degradation instance of the dynamic simulation of the same engine population and consists of multi-sensor measurements. For each cycle of a degradation instance, 21 sensor measurements, as listed in Table 1, were recorded. The multi-sensor data was contaminated with noises and each engine started with different initial health conditions and manufacturing variations, which was unknown. With limited knowledge about the true physical model, we relied solely on the multi-sensor data from the training and testing engines to understand the engine degradation process. The data can be downloaded from NASA data repository [38].

In particular, the data set FD001 that contains 100 training and testing engines is used in this study. The multi-sensor measurements for each training engine were collected until failure, whereas the multi-sensor measurements for each in-service unit were truncated at some random point before its failure. Although these engines were simulated under one operational condition and one failure mode, the engine with multiple usage conditions and failure modes may also be analyzed using the proposed method with necessary processing as in [20,39].

### 4.2. Results and Analysis on Informative Sensor Selection

Before evaluation and selection, a rough screening of the 21 sensors tells that the sensors T2, P2, P15, epr, farB, htBleed, Nf_dmd, and PCNfR_dmd are of binary or constant value and are excluded. With the remaining 13 sensors of the 100 training engines, the discussed four metrics and the two metrics of Pro and trendability (Tre) in [24] are calculated as in Table 2. For metrics of Mon, Corr, and Rob that are defined on sensors from one engine, statistics of the mean and standard deviation (std) are obtained based on results of the 100 engines. From the results, four sensors Nf, Nc, NRf, and NRc were found to be of significant difference from the other nine sensors.

**Figure 2:**
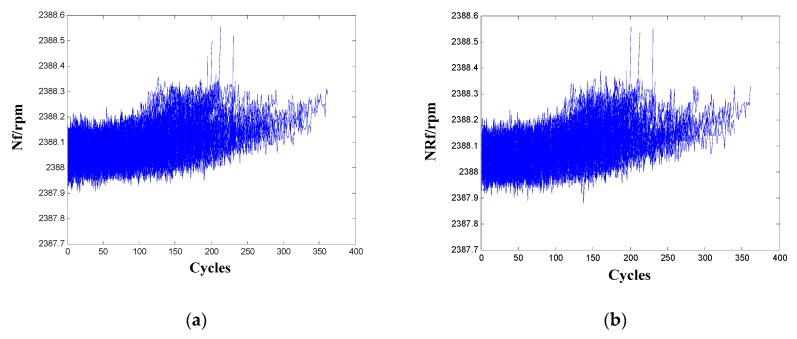
Sensor signals for the 100 training engines: (**a**) Nf; (**b**) NRf.

For the two sensors Nf and NRf, they have high variation in metric Corr and relative low value in metric Pre. Thus, these two sensors should not be selected as the informative sensors based on the analysis in Section 3.1. In addition, the two sensors score low values of metric Pro proposed in [24], verifying the effectiveness of the proposed metric Pre. Nevertheless, the metric Tre proposed also in [24] to characterize the trendability of a population of sensors scores both high for the two sensors. To make sense of interpretation, signals of the two sensors for the 100 training engines are shown in Figure 2. It shows that the two sensors are not correlated well with the usage of the engine and their ranges are both very small (actually less than 0.5) from start to failure, which validates these sensors have high variation in metric Corr and score low value in metric Pre and Pro. In previous studies using the C-MAPSS data, including [9,20], the above two sensors were also wiped out as non-informative sensors.

**Figure 3:**
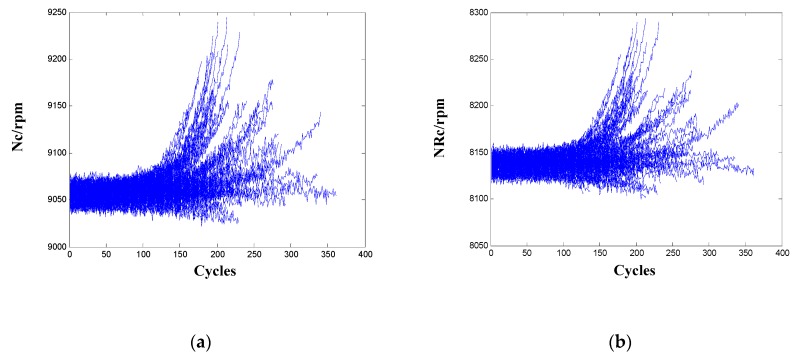
Sensor signals for the 100 training engines: (**a**) Nc; (**b**) NRc.

From the results in Table 2, the sensors Nc and NRc have high variations in both metric Mon and Corr, and their Pre scores are even lower. Also, the Pro and Tre scores of the two sensors are relatively lower as compared to other sensors. Thus, the two sensors also should not be selected as informative sensors. To interpret the evaluation results, signals of the two sensors for the 100 engines are given in Figure 3. It is observed that the two sensor signals demonstrate a very inconsistent degrading trend for the population of training engines that operate under the same condition and with one failure mode. There are increasing, decreasing, as well as oscillating patterns in the two sensors, which explains the high variations in metric Mon and Corr, as well as the low scores in metric Pre, Pro, and Tre.

For the three metrics Pre, Pro, and Tre that consider the trend consistency of a sensor for a population operate under the same condition and with one failure mode, some comparisons can be made from Table 2. The metric Tre in [24] is effective to sift out sensors without a consistent trend for the population of engines by a lower score, it cannot sift out the small range sensor, whose correlation with the degradation is poor. The proposed metric Pre and the metric Pro in [24] are both effective for wiping out these two kinds of sensors by evident lower scores. Furthermore, the Pre punishes the first kind of sensors with a lower score than that of the Pro, thus it is much easier to identify non-informative sensors with the proposed Pre. Sensors for the training engines from FD003 with the same failure mode as FD001 (i.e., HPC failure) are also evaluated and the results are in Table 3. Similar results can be drawn as those of FD001, which further shows the validity of the proposed metric Pre.

**Figure 4:**
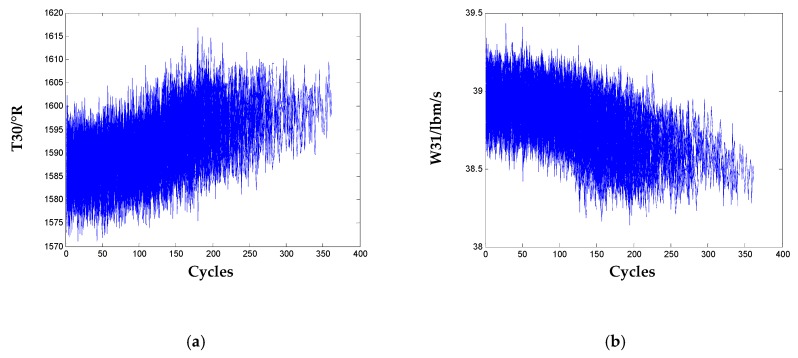
Sensor signals for the 100 training engines: (**a**) T30; (**b**) W31.

From Table 2, there is no significant difference for the evaluation results of the other nine sensors, so they are selected as the informative sensors for the HI construction of the engines. To contrast with the non-informative sensors, signals of two selected sensors are illustrated in Figure 4.

### 4.3. Results and Analysis on Degradation Modeling

**Figure 5:**
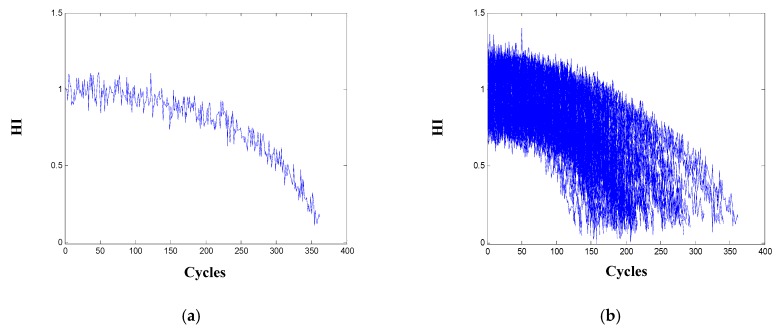
Health index (HI) for: (**a**) one engine; (**b**) all the 100 training engines.

With the selected nine-dimensional informative sensors, HI that describes the degradation of the engine is constructed as Equation (12). Data matrices for healthy and failed states of the engine are respectively created using the nine-sensor measurements of the initial five cycles and the failure cycle of all the engines. The HI of one engine and all the training engines are displayed in Figure 5. It is observed that the HI of all the engines are with the same decreasing trend and roughly vary between 1 and 0, which means a smaller HI relates to a less healthy condition of the engine.

**Figure 6:**
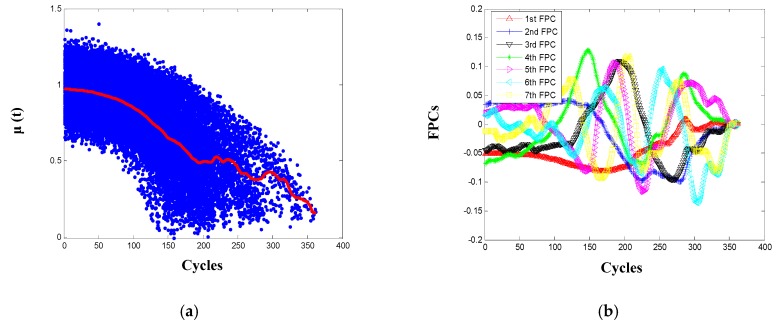
Estimation results: (**a**) the mean function; (**b**) the first seven functional principal components (FPCs).

Based on the HI of the 100 training engine systems, parameters of the FPCA-based degradation model can be estimated as stated in Section 3.3 and the main ones are shown in Figure 6. From Figure 6a, it is observed that the estimated mean function adaptively captures the general degrading trend of the 100 engines population. To ensure that sufficiently variance is retained in the truncated expansion as in Equation (19), the threshold η is set as 0.95 and the truncated terms K is 7 using Equation (7). That is, the first seven FPCs are needed to explain more than 95% of the variations of the engine degradation process, and each of the FPCs reflects one distinct varying mode of the engine deterioration process. After modeling by the FPCA method, the background health information of the 100 engines is abstracted into few parameters, i.e., the estimated mean function and the first seven FPCs, which are the common characteristics shared by engines from the training population, and the corresponding FPC scores specify the degradation peculiarity of one specific engine.

**Figure 7:**
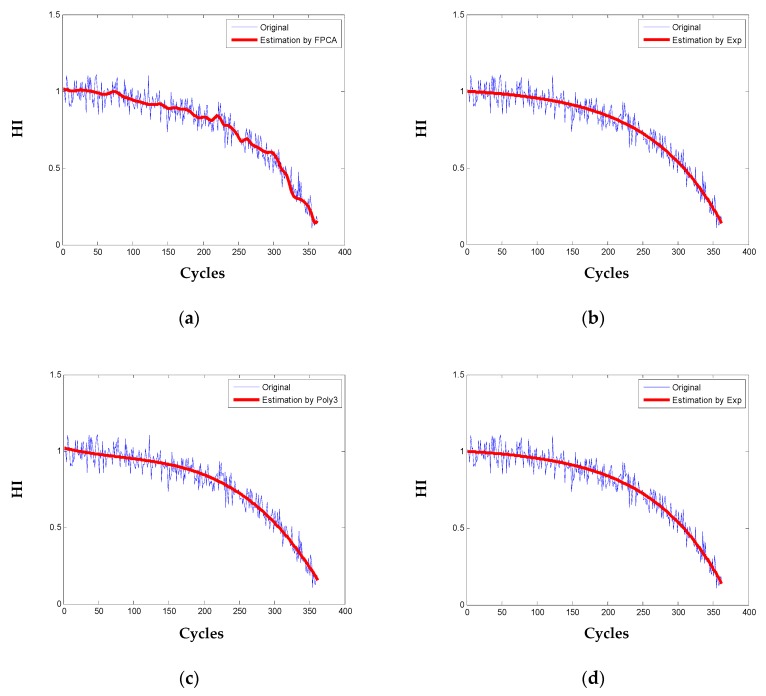
Degradation modeling for one engine: (**a**) the proposed functional principal component analysis (FPCA); (**b**) the fitting by Exponential; (**c**) the fitting by 3rd order polynomial; (**d**) the fitting by Power law.

To demonstrate the performance of the proposed method, the true parametric model of exponential (Exp) y(t) = a × exp(bt) + c [37], power law (Pow) as in [17] and 3rd polynomial (Poly3) as in [20] are also used to fit the HI of the simulated engines. Degradation modeling results for one engine are shown in Figure 7. It can be observed that the FPCA method and the three parametric models fit the degradation pattern well, but the proposed degradation model in Equation (19) reveals more local degradation dynamics of the engine. For quantitative comparison, sum of square error (SSE), the coefficient of determination (R-square) [40], and root mean square error (RMSE) are statistically calculated for the 100 engines as in Table 4.

Results in Table 4 show that the proposed method performs better than the Pow and Poly3 models, and demonstrates comparable performance to the true Exp model. However, the proposed method models the degradation adaptively without any assumption on the parametric form of the degradation pattern. This will be more significant when little knowledge is known about the latent degradation trend. Also, all the 100 engine degradation observations from the training set are pooled to estimate the parameters of the proposed degradation model. While in the parametric models, fittings are carried out with one individual engine independently, common information about the degradation process of the engine population is prone to be lost. In the following, the FPCA method is combined with Bayesian inference to predict the aero-engine health and comparison are made with the true Exp degadation model.

### 4.4. Results and Analysis on Health Prognostics

For validation of the presented method for long-term health prognostics, the multiple sensor data of the 100 testing engines from FD001 is processed following the same procedures of the training engines as detailed above. 

**Figure 8:**
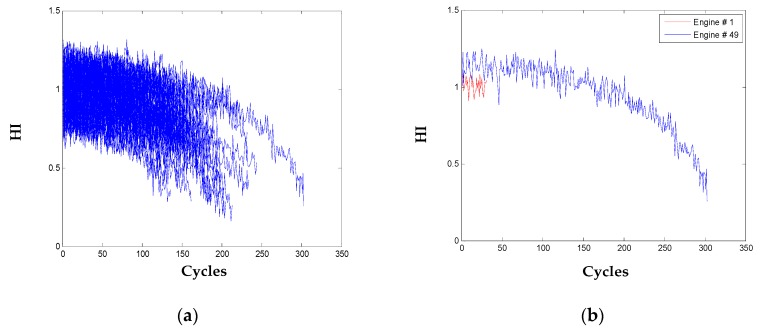
HI for: (**a**) all the 100 testing engines; (**b**) two engines.

Through linear transformation of the nine selected informative sensors with the coefficients learned from the training engines, the HI of all the testing engines and of two engines are shown in Figure 8. With the operation of one engine, its health condition degrades and the HI declines as the same pattern of the training engines. 

With the estimated results from the training engines as the prior distribution *p*_0_(**ε**) for FPC scores **ε** = (*ε*_1_, *ε*_2_, ..., *ε*_7_)*^T^*, the **ε** of a testing engine is updated to a posteriori distribution *p*(**ε**|**y**_1:*H*_) with its latest HI observations **y**_1:*H*_ using Equation (21) as discussed in Section 3.4. Statistically, the 100 testing engines are truncated from about 20% to 96% of their true useful lives. In the following, the testing engines with enough observations are chosen to demonstrate the prognostic inference. 

**Figure 9:**
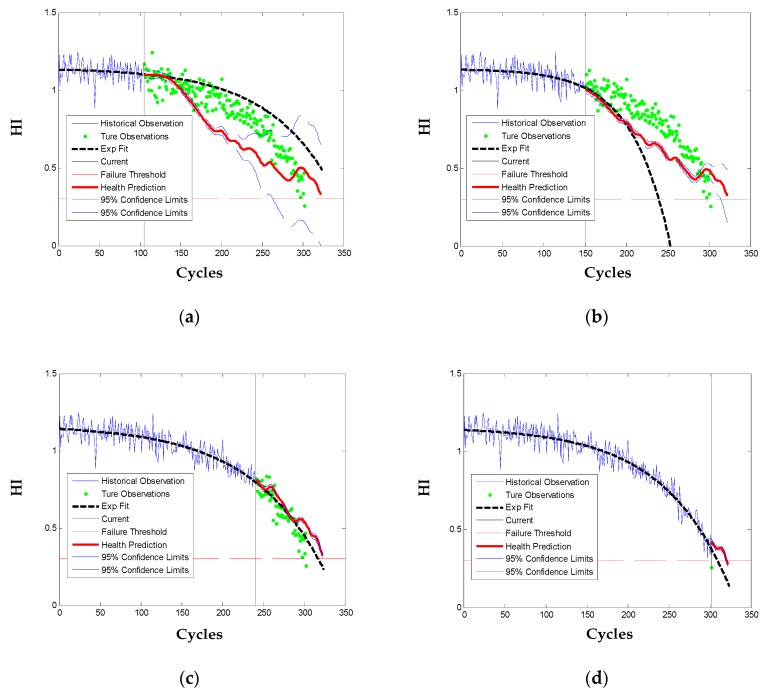
HI prediction for the testing engine # 49 at four percentages of the observations: (**a**) 35%; (**b**) 50%; (**c**) 80%; (**d**) 100%.

The testing engine # 49 consumed about 96% of its true useful life (i.e., 324 cycles) before being truncated and there are in total 303 observations. Prognostics of the engine is performed at 35%, 50%, 80%, and 100% of its health monitoring history with, respectively, H = 106, 152, 242, and 303 historical observations. The health prediction results are as Figure 9. When there is not enough historical observations (in cases of 35% and 50%), the parametric model of Exp is poorly fitted and the engine long-term health is over or under estimated. While the proposed method predicts long-term health of the engine more accurately by utilization of the mean function and the first seven FPCs learned from the training engines. With the approaching of its failure (in cases of 80% and 100%), both methods track and predict the engine health degradation with high accuracy since there accumulates sufficient historical observations to fit or update the model parameters. Also note that the proposed method reveals more local dynamics of the engine degradation, which is beneficial for explaining of the degradation variation. 

Besides the testing engine # 49, health of the other nine testing engines which are observed to consume more than 90% of their true useful lives are also predicted at four monitoring phases (i.e., 35%, 50%, 80%, and 90%) of their health monitoring histories. The HI prediction RMSEs for all the 10 engines are statistically compared in Table 5. It can be observed that the proposed method outperforms the true Exp model in the very long-term prediction, when the engines are in the early phase of service and only 35% of observations are used. With such limited historical HI, some engines are even wrongly fitted, such as # 20 and # 34. For the case of 50% observations, the proposed method also predicts better, except for engine # 20 and # 81. When the engines run into their later stages (in cases of 80% and 90%), performance of the proposed method is comparable to the true Exp model as there are sufficient HI observations to fit the parametric model.

**Figure 10:**
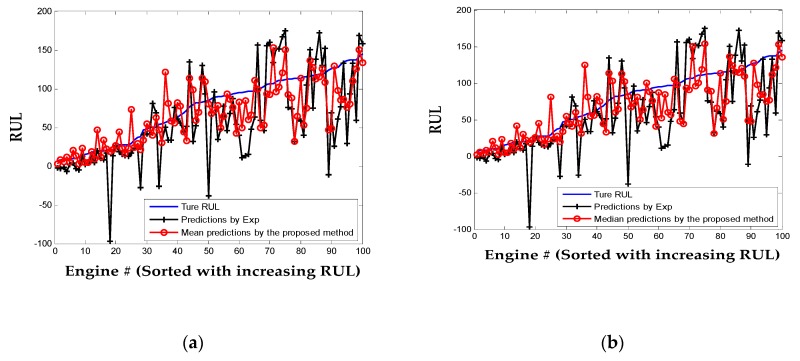
Remaining useful life (RUL) predictions for the 100 testing engines: (**a**) the mean by the proposed method; (**b**) the median by the proposed method.

To further illustrate the RUL prediction ability of the proposed approach, HI of the 100 testing engines are predicted with FPC scores updated by the last recorded observations and are extrapolated to the pre-defined failure threshold (i.e., HI*_f_* = 0.3) to estimate the final failure cycles. Results of the 100 testing engines are plotted in Figure 10. For the proposed statistical method, both the mean and median of 100 simulations are given. For the engine whose HI cannot be properly fitted to the Exp model, its life is estimated as 206 cycles, which is the mean life of the 100 training engines. It can be observed that the predicted RULs by the proposed method and Exp model are close to the actual values in the region where the RUL value is small. That is because when the engine is working closer to the failure, the degradation is enhanced and can be captured for better prognostics. However, when the engines are far from failure, the mean and median prediction results of the proposed method are more accurate than the parametric Exp model.

The common score used to evaluate the performance of prognostic method for the C-MAPSS data set as Equation (25) and prediction RMSE are also calculated and listed in Table 6. The far lower score of the proposed method (both mean and median) as well as the smaller RMSE indicate that the performance of the proposed prognostic method is better than the Exp model. Also, narrower prediction errors range and a higher number of correct-predictions are achieved using the proposed method. From the above analysis, the proposed adaptive method is more robust than the parametric Exp method, especially when one engine is in an incipient service stage of its life.

(25) Score=∑i=1nSi,Si={exp(−di13)−1, when di<0exp(di10)−1, when di≥0

where *d_i_* = predicted RUL of the *i*-th engine—true RUL of the *i*-th engine (*i* = 1, 2, ..., *n*).

## 5. Discussion

In this paper, a data-driven statistical prognostic approach for informative sensor selection and adaptive degradation modeling based on FPCA is introduced. Effectiveness of the proposed method is validated by the case study on an aircraft engine simulation experiment. Nevertheless, some key issues should be tackled for application of the presented method to real engines and other systems.

For the proposed approach to be effective for real engines, enough run-to-failure sensor data should be provided, and this is a common requirement for a data-driven approach. In real aircraft engine operation, the degradation of one engine usually begins after one long normal working stage, the so-called delay-time phenomenon. Modeling of the potential-to-failure stage is of more significance. Thus, it is better to apply the proposed method upon the detection of incipient faults. For few instances of real engine failures, and that is often the case, the jointly Gaussian assumption may no longer hold for the parameter estimation. To partially tackle this problem, some kind of bootstrap methods can be useful. As for the extension of the proposed method, especially the adaptive degradation modeling by FPCA, to monitor other systems, efforts should be put especially to the construction of a HI that relates to the degradation of these systems, as the linear data transformation method used in this paper may not be applicable.

## 6. Conclusions

To model and track the complex degradation pattern of aircraft engines for accurate and efficient prognosis, a novel method for informative sensor selection and adaptive degradation track is proposed in this paper. The deterioration sensitive sensors can be selected by the presented metrics and be fused to construct a health index that describes the degradation of an aircraft engine. Taking the degradation index of one engine as functional data, the degradation process of the engine population is then adaptively modeled by the FPCA, and future health is predicted with Bayesian inference. Experimental studies are performed on the sensor dataset of aircraft gas turbine engines, and the results verify that the proposed method can effectively select the informative sensors to model and predict the complex degradation process of the aircraft engine. The failure threshold is set to a fixed value in this study; however, large variation of the failure value requires a random failure threshold to be pursued in the future.

## Figures and Tables

**Table 1:** Sensor measurements of the aircraft engine.

Symbol	Description	Units
T2	Total temperature at fan inlet	°R
T24	Total temperature at LPC outlet	°R
T30	Total temperature at HPC outlet	°R
T50	Total temperature at LPT outlet	°R
P2	Pressure at fan inlet	Psia
P15	Total pressure in bypass-duct	Psia
P30	Total pressure at HPC outlet	Psia
Nf	Physical fan speed	Rpm
Nc	Physical core speed	Rpm
epr	Engine pressure ratio (P50/P2)	--
Ps30	Static pressure at HPC outlet	Psia
phi	Ratio of fuel flow to Ps30	pps/psi
NRf	Corrected fan speed	Rpm
NRc	Corrected core speed	Rpm
BPR	Bypass Ratio	--
farB	Burner fuel-air ratio	--
htBleed	Bleed Enthalpy	--
Nf_dmd	Demanded fan speed	Rpm
PCNfR_dmd	Demanded corrected fan speed	Rpm
W31	HPT coolant bleed	lbm/s
W32	LPT coolant bleed	lbm/s

**Table 2:** Evaluation results of the sensors for the training data set of FD001. Abbreviations: Mon, monotonicity; Corr, correlation; Rob, robustness; Pre, predictability; Pro, prognosability; Tre, trendability.

Symbol	Mon		Corr		Rob		Pre	Pro	Tre
	Mean	*std*	Mean	*std*	Mean	*std*			
T24	0.0715	*0.039* *7*	0.8365	*0.0391*	0.9997	*2.1 × 10^−^* ^5^	0.8048	0.8048	0.9679
T30	0.0515	*0.039* *5*	0.8241	*0.0397*	0.9982	*1.1 × 10^−^* ^4^	0.7619	0.7624	0.9709
T50	0.0929	*0.0456*	0.8751	*0.0294*	0.9980	*1.5 × 10^−^* ^4^	0.8709	0.8709	0.9642
P30	0.0756	*0.0533*	0.8660	*0.0382*	0.9995	*3.0 × 10^−^* ^5^	0.8381	0.8381	0.9660
Nf	0.1517	*0.0554*	0.7986	***0.1639***	1.0000	*6.3 × 10^−^* ^5^	**0.654** **3**	**0.6543**	**0.9667**
Nc	0.1482	***0.1137***	0.7535	***0.1797***	0.9997	*2.2 × 10^−^* ^5^	**0.2632**	**0.3250**	**0.919** **8**
Ps30	0.1317	*0.0531*	0.8818	*0.0262*	0.9985	*8.9 × 10^−^* ^5^	0.8671	0.8671	0.9668
phi	0.0997	*0.0494*	0.8744	*0.0312*	0.9996	*2.6 × 10^−^* ^5^	0.8466	0.8466	0.9693
NRf	0.1565	*0.0541*	0.8027	***0.1592***	1.0000	*5.2 × 10^−^* ^7^	**0.6630**	**0.6630**	**0.965** **0**
NRc	0.1574	***0.1211***	0.7623	***0.2004***	0.9997	*1.7 × 10^−^* ^5^	**0.18** **20**	**0.3106**	**0.907** **4**
BPR	0.0743	*0.0451*	0.8546	*0.0305*	0.9983	*1.0 × 10^−^* ^4^	0.8419	0.8419	0.9719
W31	0.0559	*0.0348*	0.8544	*0.0334*	0.9982	*1.2 × 10^−^* ^4^	0.8289	0.8289	0.9704
W32	0.0661	*0.0405*	0.8558	*0.0366*	0.9982	*1.1 × 10^−^* ^4^	0.8091	0.8091	0.9682

**Table 3:** Evaluation results of the sensors for the training data set of FD003 with the same failure mode of FD001.

Symbol	Mon		Corr		Rob		Pre	Pro	Tre
	Mean	*std*	Mean	*std*	Mean	*std*			
T24	0.0711	*0.0* *427*	0.8415	*0.0350*	0.9997	*2.7 × 10^−^* ^5^	0.7723	0.7723	0.9708
T30	0.0670	*0.0* *420*	0.8261	*0.0367*	0.9982	*1.0 × 10^−^* ^4^	0.7991	0.7991	0.9707
T50	0.1020	*0.0499*	0.8781	*0.0253*	0.9980	*1.0 × 10^−^* ^4^	0.8542	0.8542	0.9691
P30	0.0868	*0.0404*	0.8741	*0.0283*	0.9995	*3.0 × 10^−^* ^5^	0.8348	0.8348	0.9714
Nf	0.1545	*0.0514*	0.8334	***0.1098***	1.0000	*6.2 × 10^−^* ^7^	**0.7024**	**0.7040**	**0.9664**
Nc	0.1339	***0.1143***	0.7656	***0.1827***	0.9997	*1.8 × 10^−^* ^5^	**0.2071**	**0.2946**	**0.9198**
Ps30	0.1371	*0.0506*	0.8847	*0.0217*	0.9985	*8.9 × 10^−^* ^5^	0.8878	0.8878	0.9709
phi	0.1020	*0.0501*	0.8838	*0.0213*	0.9996	*2.8 × 10^−^* ^5^	0.8659	0.8659	0.9649
NRf	0.1530	*0.0551*	0.8256	***0.1225***	1.0000	*6.6 × 10^−^* ^7^	**0.6990**	**0.6998**	**0.9665**
NRc	0.1388	***0.1098***	0.7479	***0.2379***	0.9997	*1.9 × 10^−^* ^5^	**0.1386**	**0.2760**	**0.9183**
BPR	0.0740	*0.0473*	0.8583	*0.0305*	0.9983	*1.1 × 10^−^* ^4^	0.8487	0.8489	0.9660
W31	0.0549	*0.0368*	0.8532	*0.0376*	0.9982	*1.2 × 10^−^* ^4^	0.8388	0.8388	0.9691
W32	0.0607	*0.0351*	0.8544	*0.0330*	0.9982	*8.3 × 10^−^* ^5^	0.8280	0.8280	0.9719

**Table 4:** Comparisons of the degradation modeling results. Abbreviations: SSE, sum of square error; R-square, the coefficient of determination; RMSE, root mean square error.

Method	SSE		R-Square		RMSE	
	Mean	*std*	Mean	*std*	Mean	*std*
FPCA	0.6597	*0.1571*	0.9305	*0.0224*	0.0565	*0.0026*
Exp	0.6423	*0.1567*	0.9324	*0.0219*	0.0562	*0.0025*
Poly3	0.6673	*0.1592*	0.9210	*0.0219*	0.0570	*0.0026*
Pow	0.6736	*0.1658*	0.9194	*0.0222*	0.0575	*0.0027*

**Table 5:** Comparison of health prognostic results for the 10 testing engines.

Engine #	RMSE							
	*35%*		*50%*		*80%*		*90%*	
	Exp	FPCA	Exp	FPCA	Exp	FPCA	Exp	FPCA
20	3.4 *× 10*^4^	0.0800	0.0591	0.0880	0.0570	0.0737	0.0664	0.0910
31	0.1568	0.1345	0.9053	0.1395	0.0638	0.0954	0.0532	0.0829
34	3.4 *× 10*^3^	0.1688	1.4623	0.1506	0.0649	0.0981	0.0677	0.0921
35	0.1599	0.0818	0.1701	0.0730	0.1091	0.0813	0.0535	0.0727
42	1.5788	0.0960	0.1034	0.0971	0.0710	0.0863	0.0534	0.0659
49	0.1345	0.1149	0.9053	0.1119	0.0638	0.0752	0.0532	0.0795
68	2.4974	0.1100	0.5035	0.1246	0.0621	0.0959	0.0844	0.0962
76	20.285	0.1038	0.1593	0.1264	0.0701	0.0969	0.0594	0.0666
81	0.3026	0.1242	0.1005	0.1292	0.1672	0.1216	0.0624	0.0936
82	3.1 *× 10*^4^	0.1052	0.5006	0.1391	0.0735	0.0903	0.0671	0.0661
mean	6.8 *× 10*^3^	0.1119	0.4869	0.1179	0.0796	0.0915	0.0611	0.0807

**Table 6:** Comparison of RUL prediction results for the 100 testing engines.

Method		Score	RMSE	Rang of Prediction Errors	Under-Predictions	Correct- Predictions	Over-Predictions
**Exp**		67,352	45.40	[−135, 63]	76	1	23
FPCA	mean	3092	28.06	[−82, 65]	65	3	32
	median	3567	28.70	[−83, 68]	67	5	28
